# Supplementary figures and images for: Effect of different thresholds for CT perfusion volumetric analysis on estimated ischemic core and penumbral volumes
Source: PLoS One. 2021 Apr 21;16(4):e0249772. doi: 10.1371/journal.pone.0249772 (PMC8059822; doi:10.1371/journal.pone.0249772)

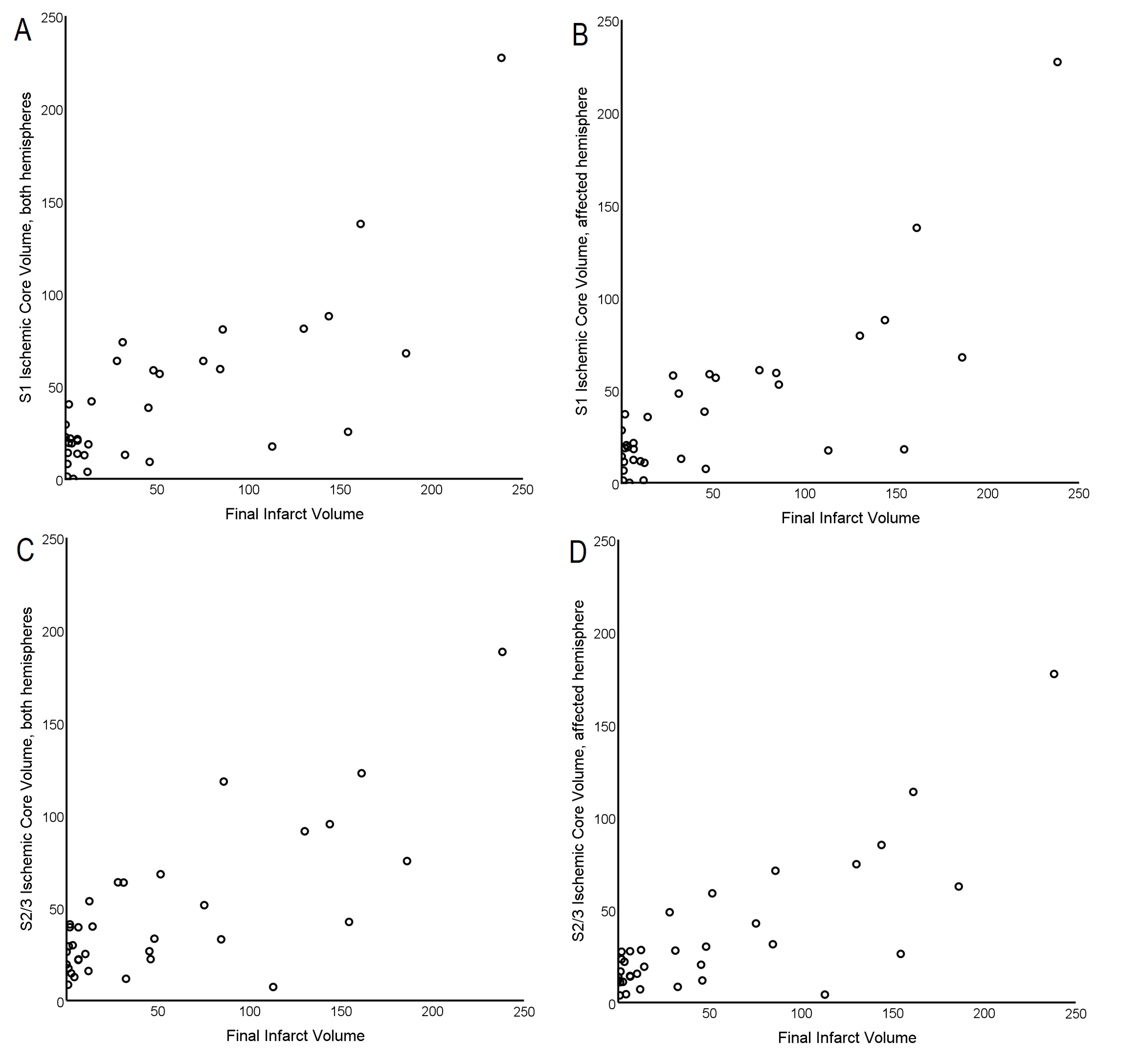

Supplement: S1 Fig — S1 = Setting 1 (panel A and B, using both hemispheres and affected hemisphere only and S2/3 = Setting 2 and 3 (Panel C and D, using both hemispheres and affected hemisphere only). (TIF) [file pone.0249772.s001.tif]
