# Supplementary material for: Effect of different thresholds for CT perfusion volumetric analysis on estimated ischemic core and penumbral volumes
Source: PLoS One. 2021 Apr 21;16(4):e0249772. doi: 10.1371/journal.pone.0249772 (PMC8059822; doi:10.1371/journal.pone.0249772)
Supplement: S1 Text — (PDF) [file pone.0249772.s002.pdf]

**S1 Text.** Depiction of endovascular procedures

EVT procedures were performed in a dedicated biplane neuroangio suite (Siemens Healthcare, Erlangen, Germany) by experienced interventional radiologists. Endovascular treatment interventions were performed primarily under conscious sedation (N=39) or local/topical anesthesia (N=8) and general anaesthesia (N=5) was adopted only if the patient was overly agitated or restless. Femoral access with an 8F/80cm introducer (Cook Incorporated, Bloomington, Indiana, USA) was utilized in all interventions, and a multipurpose guiding catheter was delivered to the relevant internal carotid or vertebral artery over a 5F diagnostic catheter (5F JR4; Cordis, Miami, FL, USA). The thrombectomy aspiration catheter (5MAX, 5MAX60, or ACE68; Penumbra Inc., Alameda, CA, USA) was selected based on the site of the vessel occlusion, and delivered to the occlusion over a microcatheter (Neuroslider21; Acandis GmbH, Pforzheim, Germany) and a 0.014 inch microwire (Synchro2; Stryker; Fremont, CA, USA or Transend EX). Thrombus was extracted either by direct aspiration (N=11) or with stent retriever (N=35) (Solitaire; ev3/Covidien, Irvine, CA, USA or Aperio; Acandis GmbH, Pforzheim, Germany). In 6 cases, reperfusion was left at TICI 0 despite the recanalizing efforts.
