# Supplementary material for: Effect of different thresholds for CT perfusion volumetric analysis on estimated ischemic core and penumbral volumes
Source: PLoS One. 2021 Apr 21;16(4):e0249772. doi: 10.1371/journal.pone.0249772 (PMC8059822; doi:10.1371/journal.pone.0249772)
Supplement: S1 Table — (PDF) [file pone.0249772.s003.pdf]

S1 Table. Excluded patients

All consecutive EVT patients with anterior circulation LAO (age ≥18 years)		125
Exclusion due to deviant image data		32
	Imaging performed elsewhere	23
	CTP not performed	5
	CTP failure	2
	Control imaging with MRI	2
Exclusion due to good angiographic response to coincident intravenous thrombolytic therapy		14
Other reason for exclusion		27
	Fluctuating symptoms	2
	Chronic Intracranial stenosis in angiography	5
	Proximal ICA stenosis requiring carotid artery stenting	20
Total excluded patients		73
Total included patients		52

Abbreviations: EVT=Endovascular treatment LAO=Large artery occlusion, CTP=Computed tomography perfusion, MRI=Magnetic resonance imaging, ICA=Internal carotid artery
