# Supplementary material for: Effect of different thresholds for CT perfusion volumetric analysis on estimated ischemic core and penumbral volumes
Source: PLoS One. 2021 Apr 21;16(4):e0249772. doi: 10.1371/journal.pone.0249772 (PMC8059822; doi:10.1371/journal.pone.0249772)
Supplement: S2 Table — (PDF) [file pone.0249772.s004.pdf]

S2 Table. Patient baseline and admission imaging characteristics

All patients, n		52
Age, years, mean ± SD		68.3 ± 9.3
Female, n (%)		22 (42.3)
Atrial fibrillation, n (%)		25 (48.1)
Hypertension, n (%)		34 (65.4)
Coronary artery disease, n (%)		11 (21.2)
Hyperlipidemia, n (%)		19 (36.5)
Diabetes, n (%)		12 (23.1)
Heart failure, n (%)		13 (25.0)
Age over 80 years, n (%)		6 (11.5)
Location of Clot/occlusion, n (%)	proximal ICA	13 (25)
	M1 segment, MCA	26 (50)
	M2 segment, MCA	13 (25)
Left hemisphere n (%)		28 (54)
Safe onset n (%)		35 (67.3)
Unknown onset n (%)	Total	17 (32.7)
	Last symptom free	4 (7.7)
	Wake-up stroke	7 (13.5)
	Found/unknown	6 (11.5)

Abbreviations: IQR=Interquartile range, SD=Standard Deviation, ICA=Internal Carotid Artery, MCA=Middle Cerebral Artery,
