# Supplementary material for: Effect of different thresholds for CT perfusion volumetric analysis on estimated ischemic core and penumbral volumes
Source: PLoS One. 2021 Apr 21;16(4):e0249772. doi: 10.1371/journal.pone.0249772 (PMC8059822; doi:10.1371/journal.pone.0249772)
Supplement: S3 Table — (PDF) [file pone.0249772.s005.pdf]

**S3 Table.** Procedural and outcome characteristics

| | | |
|---|---|---|
| Preoperative Iv Thrombolysis n (%) | | 31 (59.6) |
| Form of sedation/anesthesia n (%) | Conscious sedation | 39 (75.0) |
| | General anesthesia | 5 (9.6) |
| | Topical anesthesia | 8 (15.4) |
| Safe onset to reperfusion (min), median (IQR), (n=28) | | 290 (206-354) |
| Safe onset to imaging (min), median (IQR), (n=35) | | 159(91-242) |
| Imaging to reperfusion time (min) median (IQR) (n=80) * | | 113 (90-155) |
| | Under median time n (%) | 21 (40.4) |
| | In median time or over or no reperfusion n (%) | 31 (59.6) |
| TICI Score | TICI 2b/3 n (%) | 34 (65.4) |
| | TICI 0-2a, n (%) | 18 (34.6) |
| Intra-arterial procedure | Retriever n (%) | 35 (67.3) |
| | Aspiration only n (%) | 11 (21.2) |
| | Clot not reached n (%) | 6 (11.5) |
| Follow-up CT delay (days), mean ± SD | | 1 ± 0.75 |
| FIV seen on Follow-up CT (mL) median (IQR) | | 26.1 (6.4-82.2) |
| Infarct Size over 70 mL n (%) | | 14 (26.9) |

*11 Patients with TICI 0 excluded. Abbreviations: IQR=Interquartile Range, CTP=Computed Tomography Perfusion, TICI=Thrombolysis in Cerebral Infarction Scale, SD=Standard deviation, CT=Computed Tomography. IC=Ischemic Core, FIV=Final Infarct Volume
