# Supplementary material for: Effect of different thresholds for CT perfusion volumetric analysis on estimated ischemic core and penumbral volumes
Source: PLoS One. 2021 Apr 21;16(4):e0249772. doi: 10.1371/journal.pone.0249772 (PMC8059822; doi:10.1371/journal.pone.0249772)
Supplement: Attachment — Submitted filename: Response to Reviewers.pdf [file pone.0249772.s007.pdf]

07.03.2021

Dear Editors,

We thank the Reviewers for their valuable comments on the manuscript “Effect of different thresholds for CT perfusion volumetric analysis on estimated ischemic core and penumbral volumes” (PONE-D-20-23892). Additional changes have now been made to the manuscript according to the feedback.

We have fixed the issues presented by each reviewer and hope that our manuscript is now suitable for publication in PLOS ONE.

Best regards,

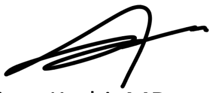

Simo Karhi, MD,

On behalf of all authors.

Response to Reviewer #1:

The authors responses to the comments and criticism were reasonable and the manuscript is updated appropriately. There is a minor error in the reference section. The items #27 and 36 refer to the same manuscript (Campbell BC, et al).
The authors are thankful for the excellent feedback and comments giving us new viewpoints to our study and manuscript and also for future research on the subject. The error in the reference section has now been fixed.

Response to Reviewer #2: *CTP thresholds are used to select stroke patients for EVT. In this revised paper the authors show wide disparities in infarct core and penumbral volumes when different software packages are used. The primary outcome is the accuracy of CTP thresholds with the final infarct volume measure by CT. There remain several important concerns that the authors' need to address:*

The authors wish to point out that the study compares the results produced by a single software package using three different threshold levels.

"1) I would recommend the comparisons between the CTP thresholds be a second aim and not merely added in the supplement. The abstract, methods, results and discussion need to reflect this change."
Thank you for the valuable comments. To address the Reviewer's concern, the comparison between the three CTP threshold settings has now been mentioned as the second aim of the study in Introduction, and furthermore, the Abstract and Discussion have been modified to better reflect the importance of the differences between the CTP thresholds. The suggested supplementary Bland Altman Plots have now been transferred from the supplementary materials to be part of the main manuscript in the Results section.

"2) The Bland Altman plots comparing CTP thresholds are incomplete. Typically, a BA plot shows the mean difference, 1.96 SD around that difference and the upper and lower margins. In the supplementary figure plots B and C are a duplicate."
Based on the feedback, one of the two Bland Altman plots visualizing the setting pair S1 vs S2 when comparing Ischemic Core Volumes has now been excluded due to the similarities between the original panels B and C.<br><br>As requested, the Bland Altman Plot figure now includes the numeral explanation for the lines for mean difference, and the 1.96 SD around the difference. Upper and lower margins for mean difference are now visualized with additional thinner dotted lines.<br><br>In addition, an alternative and more detailed figure 2 (visualizing also upper and lower 95% confidence limits for the upper and lower "agreement limits") is included based on a consultation of our biostatistician (see uploaded file titled: Alternative Fig 2). We are willing to use either of these alternative plots according to the Reviewer's and Editor's preferences.

"3) When measuring the accuracy of CTP thresholds for core and penumbra with FIV typically a sensitivity analysis is performed not Pearson correlation."
Thank you for the comment. The use of sensitivity analysis was considered by the authors when choosing between different methods for statistical analysis. Among these methods, Pearson Correlation was selected and has also been used in earlier literature, for example: *Austein F, Riedel C, Kerby T, et al. Comparison of perfusion CT software to predict the final infarct volume after thrombectomy. Stroke. 2016; 47:2311–2317.*
